# Live Cervical Ectopic Pregnancy in a Patient With Previous Cesarean Section: A Case Report With Review of Literature

**DOI:** 10.7759/cureus.36940

**Published:** 2023-03-30

**Authors:** Ameen Ansari, Avinash Dhok, Suruchi Dhawan, Prashant Onkar, Priya Potdukhe, Kajal Mitra

**Affiliations:** 1 Radiodiagnosis, NKP Salve Institute of Medical Sciences (SIMS) and Lata Mangeshkar Hospital, Nagpur, IND

**Keywords:** cervical pregnancy, previous cesarean section, endovaginal ultrasonography, ectopic pregnancy, live cervical pregnancy

## Abstract

Live cervical ectopic pregnancy is a rare type of ectopic pregnancy and accounts for <1% of all ectopic pregnancies. Prompt diagnosis and early management with systemic or local administration of methotrexate is the treatment of choice in most cases. If the pregnancy is complicated, it can lead to significant hemorrhage, which may require a hysterectomy to save the life of the patient. We report a case of live cervical ectopic pregnancy in a 26-year-old patient with a history of previous cesarean section and presenting with silent bleeding per vaginum for six hours.

## Introduction

For pregnant patients with pelvic pain, ultrasonography is the investigation of choice because it is readily available, noninvasive, and safe, and does not require special preparation [1]. An ectopic pregnancy occurs when a growing blastocyst implants itself somewhere else other than the endometrium [2]. Any extra-endometrial gestation is called an ectopic pregnancy, and in cervical pregnancy, the product of conception implants in the endocervical canal [3]. Early detection of unruptured ectopic pregnancy also enables the adoption of a less invasive form of therapy [4]. The reported incidence of ectopic pregnancy is 1.9% of all pregnancies [5]. Cervical pregnancy has historically been linked to significant morbidity and negative effects on the patient in the form of secondary infertility [6]. The use of intrauterine contraceptive devices, previous cesarean sections, previous endometrial curettage, and assisted reproductive techniques are some of the known risk factors [7]. Hormonal estimation and pelvic ultrasound are the main components of the initial examination of ectopic pregnancy [8].

## Case presentation

A 26-year-old female patient came to our emergency department with complaints of bleeding per vaginum for six hours, associated with nausea and 3-4 episodes of non-bilious vomiting and lower abdominal pain. The patient gave a history of amenorrhea of 1.5 months duration, with a history of cesarean section during the previous pregnancy two years back. The urine pregnancy test of the patient was also positive. The vital signs of the patient were normal.

The patient’s other routine laboratory investigations were normal but were immediately referred to an obstetrician, where ultrasonography of the pelvis was recommended.

**Figure 1:**
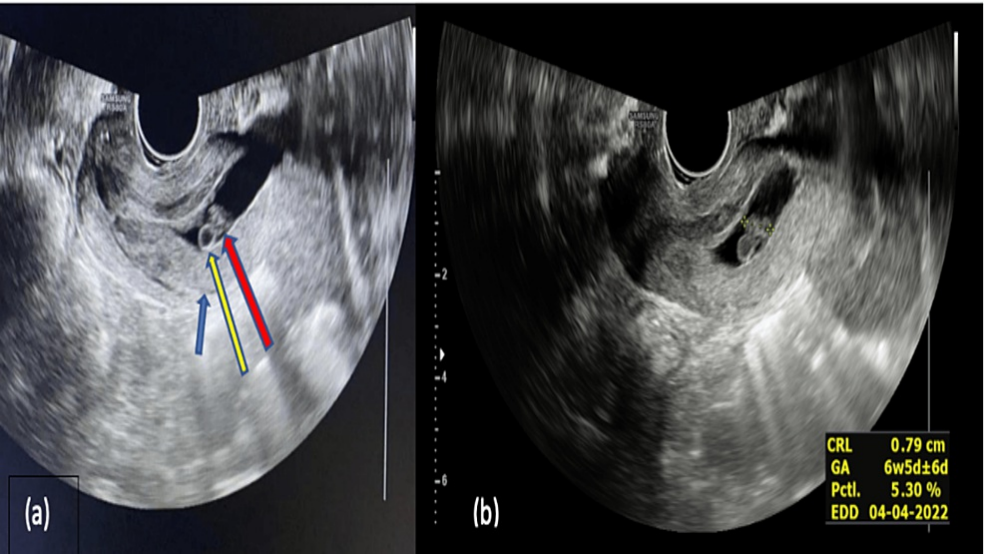
Endovaginal ultrasonography image showing the (a) cervix (blue arrow) with yolk sac (yellow arrow) and fetal pole (red arrow) within the endocervical canal, with (b) CRL of 7.9 mm corresponding to six weeks and five days of gestation. CRL: crown-rump length

**Figure 2:**
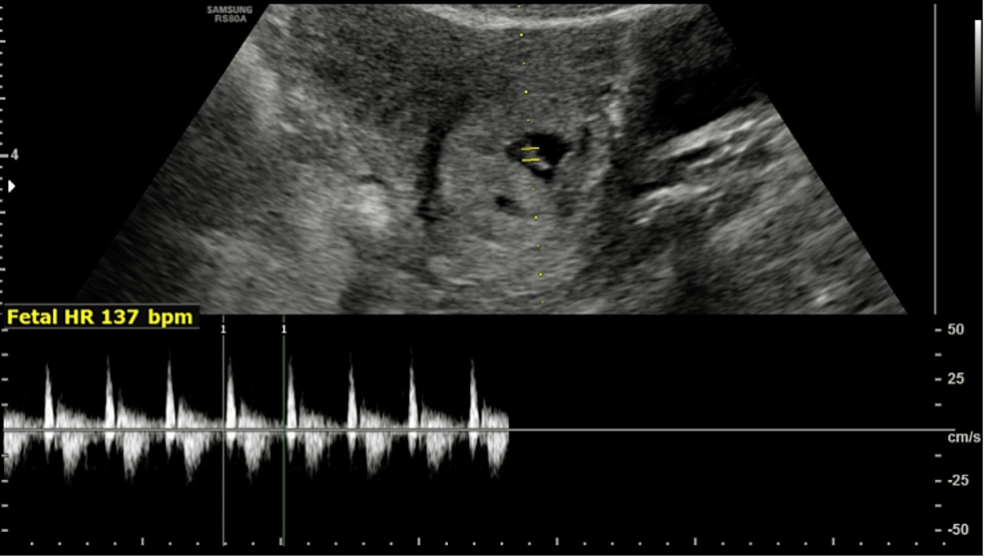
Transabdominal ultrasonography with spectral Doppler showing cardiac pulsations of 137 bpm. bpm: beats per minute, HR: heart rate

On endovaginal ultrasonography, a single gestational sac was seen within the endocervical canal with a crown-rump length (CRL) of 7.9 mm corresponding to a gestational age of six weeks and five days (Figure 1) along with fetal pole and cardiac pulsations within it (Figure 2).

**Figure 3:**
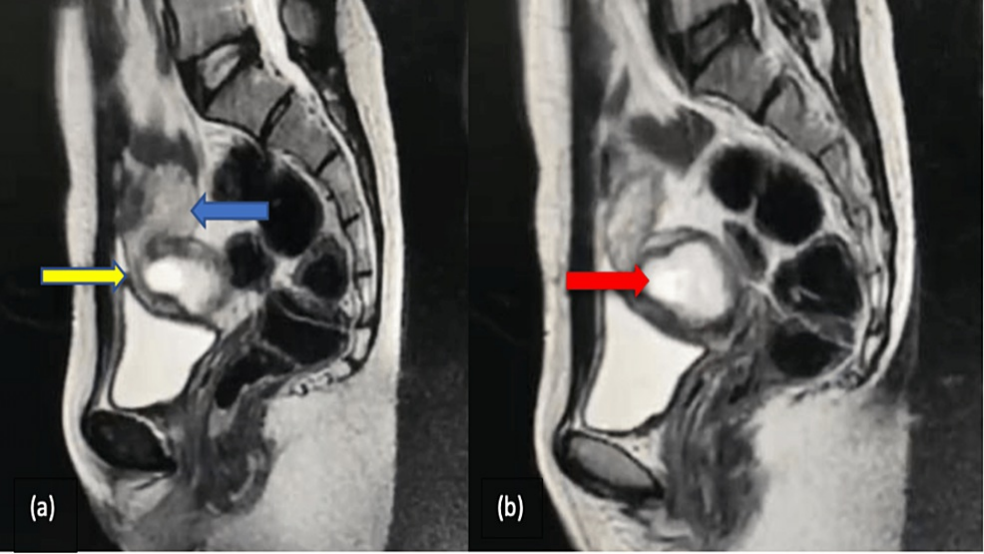
Sagittal T2-weighted MRI showing the (a) uterus (blue arrow) and cervix (yellow arrow), and (b) an irregular gestational sac (red arrow) measuring 4.0 × 2.8 × 3.4 cm is noted in the endocervical canal distal to the internal os appearing hyperintense with an incomplete circumferential hypointense rim. MRI: magnetic resonance imaging

The patient was further subjected to magnetic resonance imaging (MRI) to rule out any other pelvic pathology. The MRI confirmed the location and extent of the lesion (Figure 3). No adnexal abnormality was demonstrated on MRI.

**Figure 4:**
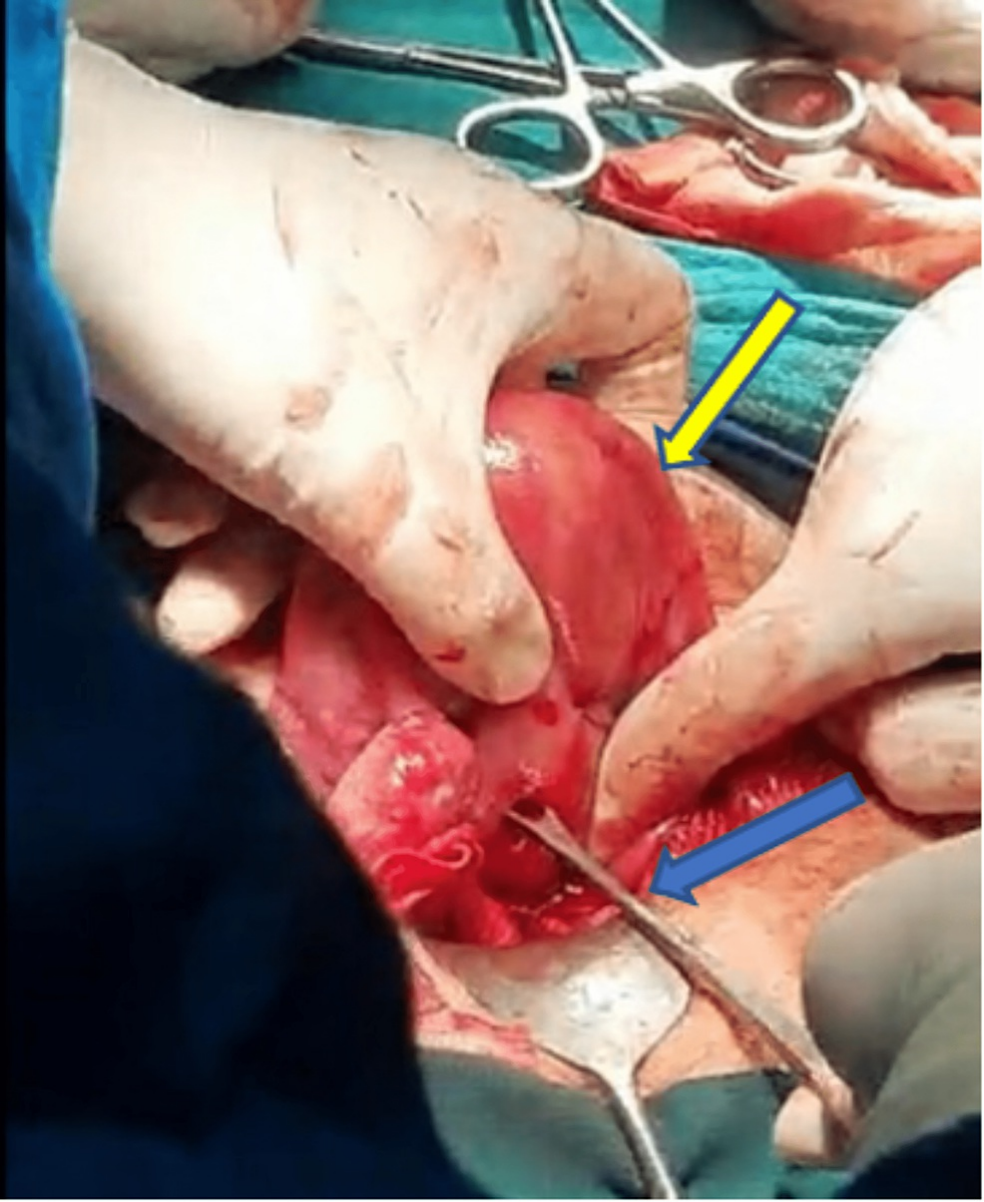
Intraoperative photograph of the uterus (yellow arrow) confirming the diagnosis on curettage with ovum forceps (blue arrow).

The patient was sent for emergency laparotomy, suction and curettage was done using ovum forceps, and the products of conception were taken out (Figure 4).

## Discussion

Although faster detection and treatments have dramatically reduced maternal mortality over the past 20 years, ectopic pregnancy is still the number one cause of mortality in pregnant women in the first trimester [8].

Cervical pregnancy is indicated by the presence of a gestational sac, visible chorionic tissue, or cardiac pulsations distal to the internal os with opened internal os and closed external os. No intrauterine endometrial gestational sac will develop, and the uterus will take an “hourglass” shape with an expanded cervix [7].

In patients with nonviable pregnancies, the ability to distinguish between a real cervical pregnancy and an incomplete abortion depends on visualization of the closed internal os. Due to the increased thickness and echogenicity of the endometrium during pregnancy, it may be difficult to assess the internal os [9].

In cases where the diagnosis is uncertain, MRI may be helpful [10].

Because of the increased usage of intrauterine contraceptive devices, pelvic inflammatory diseases, sexually transmitted diseases, and assisted reproductive technologies, the incidence of ectopic pregnancies is increasing [2].

Thanks to endovaginal ultrasonography for early detection of cervical pregnancy and benefiting patients by improving diagnosis. The gestational sac, endometrium, and adnexa all can be well-evaluated [6].

Because the cervix lacks contractile tissue, patients with cervical ectopics frequently bleed heavily [1].

Historically, hysterectomy had been used to treat cervical pregnancy; however, patients with non-ruptured ectopic pregnancy may benefit from less invasive procedures, medicinal care with methotrexate, and expectant management [5].

When the chorionic gonadotropin level of a pregnant woman is less than 2,000 mIU/mL and her chorionic gonadotropin doubling time indicates a nonviable pregnancy, suction curettage plays a vital role in the management of ectopic pregnancy [4].

## Conclusions

Live cervical ectopic pregnancies are relatively uncommon; however, increased cases are being reported because of factors such as high cesarean section rate and increased usage of assisted reproductive techniques for the management of infertility. The most common presenting symptom is painless vaginal bleeding, but sometimes, massive bleeding can occur, needing a hysterectomy to save the life of the patient. Endovaginal ultrasonography serves as an excellent modality in the early diagnosis of cervical ectopic pregnancy.
